# A dataset of global variations in directional solar radiation exposure for ocular research using the libRadtran radiative transfer model

**DOI:** 10.1016/j.dib.2023.108926

**Published:** 2023-01-21

**Authors:** Maxime Durand, Andrew McLeod

**Affiliations:** a Organismal and Evolutionary Biology (OEB), Viikki Plant Science Centre (ViPS), Faculty of Biological and Environmental Sciences, University of Helsinki, 00014, Finland; b School of GeoSciences, The University of Edinburgh, Crew Building, The King's Buildings, Alexander Crum Brown Road, Edinburgh EH9 3FF, United Kingdom

**Keywords:** Radiance, Spectral composition, Eye, Ultraviolet, Albedo, Aerosols

## Abstract

The libRadtran radiative transfer model was used to calculate examples of the amount of spectral radiation (250–2500 nm) incident on the eye. Simulations were run for every hour of four individual days (representing spring, summer, autumn and winter) and at three latitudes (from southern Spain to central Finland), in order to demonstrate diurnal and seasonal variations in directional photon flux density due to solar angle. The dataset also includes outputs under strong and weak aerosol optical density, three bidirectional reflectance distribution functions (corresponding to a forested, urban and snowy ground surfaces), eight cardinal directions, and two tilt angles (either looking towards the horizon or 15° downward). All simulations were parametrized according to local meteorological conditions (elevation, pressure, temperature) and atmospheric condition on the simulated day (aerosol optical density, water column, O_3_ and NO_2_ concentrations), at 170 cm above the ground (representing the average human height). Example data are presented for a 17° field of view relevant to exposure of the macula (without correction for spectral transmission of ocular media). For each simulation, a file in “.csv” format is available containing the radiance at each wavelength. The simulations were performed in batches via R software, from a template input parameter file modified for each simulation from a summary input table. The R code and input files are also available. By describing the amount and wavelength composition of directional radiation incident on the eye, this dataset and future simulations will help parameterize research aimed at understanding and mitigating eye-related diseases.

**Specifications Table**

**Table:** 

Subject	Atmospheric Science
Specific subject area	Radiative transfer modeling the photon flux density incident on the human eye
Type of data	Table Figure
How the data were acquired	The acquired data are model outputs of the libRadtran (v2.0.3) radiative transfer model. libRadtran was run on Windows 10 using the Cygwin GNU collection (v3.1.4). Input parameters for each simulation were written in the .csv file and a new input file was automatically written, and the model ran via an R script (v4.0.3) from a template input file. For the atmospheric input data, we used the public domain data from the satellite AERONET network.
Data format	Raw Analyzed
Description of data collection	Three simulations were performed, parametrized according to local meteorological (elevation, pressure, temperature) and atmospheric conditions (aerosol optical density, water column, O_3_ and NO_2_ concentrations). The model was parametrized for a subject looking northward toward the ground (−15**°** from horizon), at 170 cm above the ground. We tested the effect of latitude, season, time of day, aerosol optical density, albedo, and viewing direction.
Data source location	Institution: University of Helsinki City/Town/Region: Helsinki, Uusimaa Country: Finland Latitude and longitude: 60.227 N, 25.018 E
Data accessibility	Repository name: Zenodo Data identification number: 10.5281/zenodo.7050989 Direct URL to data: https://zenodo.org/record/7050989

## Value of the Data

• Quantifying the properties of radiation incident on the eye is crucial to improve the understanding of eye diseases such as keratitis, cataract formation and macula degeneration.
• These simulations demonstrate the importance of directional photon flux density and how this changes with latitude in comparison with horizontal photon flux density as typically measured by global monitoring networks.
• This dataset will benefit medical research investigating eye and light-related disease, as well as atmospheric modelers interested in understanding the properties of solar radiation perceived by humans.
• These data demonstrate how modeling directional exposures can be used to investigate how atmospheric factors, diurnal and seasonal changes, solar angle, viewing direction and ground albedo affect the amount and spectral composition of radiation received by the eye. A script is also available for further user-defined simulations.
• The simulations demonstrate the efficacy of radiative transfer modeling for future applications incorporating different ocular fields of view, spectral transmission of ocular components and human behavior relevant to corneal, lenticular and macula exposure to solar radiation.

## 1 Objective

Directional solar photon flux density has particular relevance to research on eye disease such as keratitis, cataract formation and macular degeneration because ocular components (cornea, lens, retina) experience different exposures dependent on global location, structural geometry of the eye and human behavior [1,2]. Age-related macular degeneration (AMD) is one of the main eye diseases causing blindness in developed countries, likely as a consequence of increased population aging [3] and treatment is often expensive, if available at all [4]. A better understanding of the mechanisms by which such eye disease occurs is crucial if we want to optimize health care. It requires quantification of both the amount and composition of radiation received by the eye. We therefore evaluated exposure of the eye using a radiative transfer model because it allows calculation of directional spectral photon flux density under a variety of spatio-temporal conditions. It can provide a comprehensive assessment of the properties of solar radiation incident on the eye and as an example we used 17° (0.06901537 sr) field of vision (FOV) relevant to the macula [5] as an example (without correction for spectral ocular transmission).

## 2 Data Description

The dataset covers three simulations [6]. For each simulation, separate files are available for each condition (*e.g.* latitude, time, date, see Table 1). Each file includes radiance (in mW m^−2^ nm^−1^ sr^−1^) at each calculated wavelength (column name lambda, in nm). Additionally, row numbers are added to each file. Each file name specifies what were the conditions for the simulation. In order, and separated by the character “_”, the fine names describe the cardinal direction (in degree clockwise from North), the hour (0 to 23), the date (in day-month-year order, separated by a dot), and the location. For simulation 1 and 2, the aerosol optical density is given as well(0.1 or 2.5). for simulation 3, the soil surface type is added (1 for forest, 13 for urban, and 19 for snow). The folder “inputs” contains the template input file (inp_ex.inp) for libRadtran, and all the parameters added for each simulation (in the “input_Sim1–3.csv” files). An R script to write the input files and run the simulations (on Windows 10 using Cygwin) is also provided. Table 1 summarize the files and conditions used for each simulation.

**Table 1:** Summary of the parameters used for each simulation.

	Simulation 1	Simulation 2	Simulation 3	Conditions
Number of files	4608	576	5184	
Wavelength range	250 - 500 nm	250 - 2500 nm	250 - 500 nm	
Latitudes	3	3	1 (Lammi only)	Lammi, Finland - Lille, France - Murcia, Spain
Dates	4	4	4	April 17th - July 1st - September 1st - November 6th 2019
Times	24	24	24	Once per hour
Aerosol optical density	2	2	1 (0.1 only)	0.1 - 2.5
Soil surface	1 (Urban only)	1 (Urban only)	3	Forest - Urban - Snow
Cardinal direction	8	1 (North only)	9	Every 40 or 45° from North
Tilt angle	1 (−15° only)	1 (−15° only)	2	0° from horizon - 15° toward the ground

**Fig. 1:**
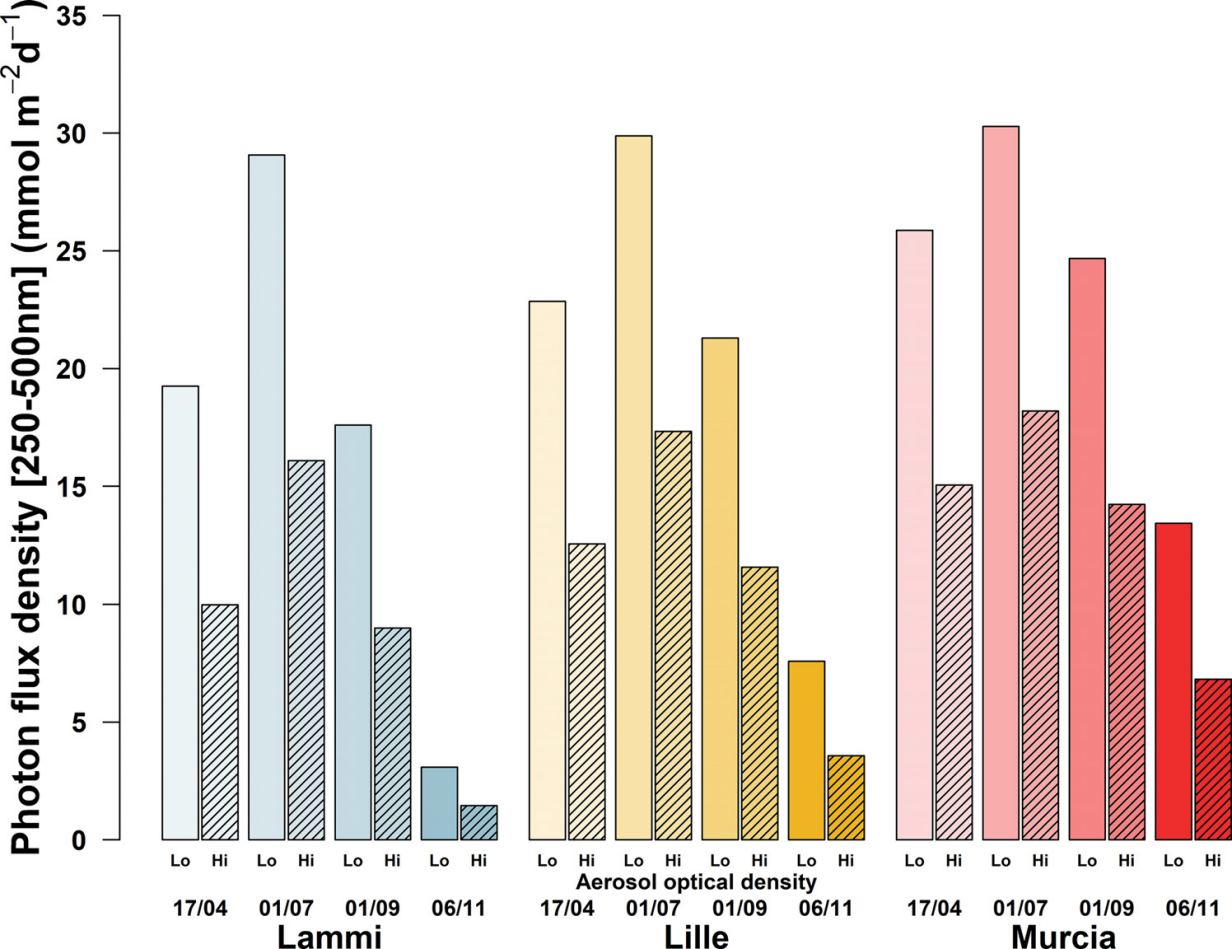
Total daily photon flux density (between 250 and 500 nm wavelength) incident on a sensor (*e.g.* an eye) looking at 15° below the horizon and at 170 cm above the ground, from three locations (Lammi, Finland; Lille, France; Murcia, Spain), four dates (April 17th, July 1st, September 1st, and November 6th 2019), and two aerosol optical densities (Hi: 2.5, Lo: 0.1). Simulations were parametrized according to local meteorological condition (elevation, pressure, temperature) and atmospheric conditions on the simulated day (water column, O_3_ and NO_2_ concentrations). Data were averaged over eight cardinal directions, and normalized to a field of view of 17°.

**Fig. 2:**
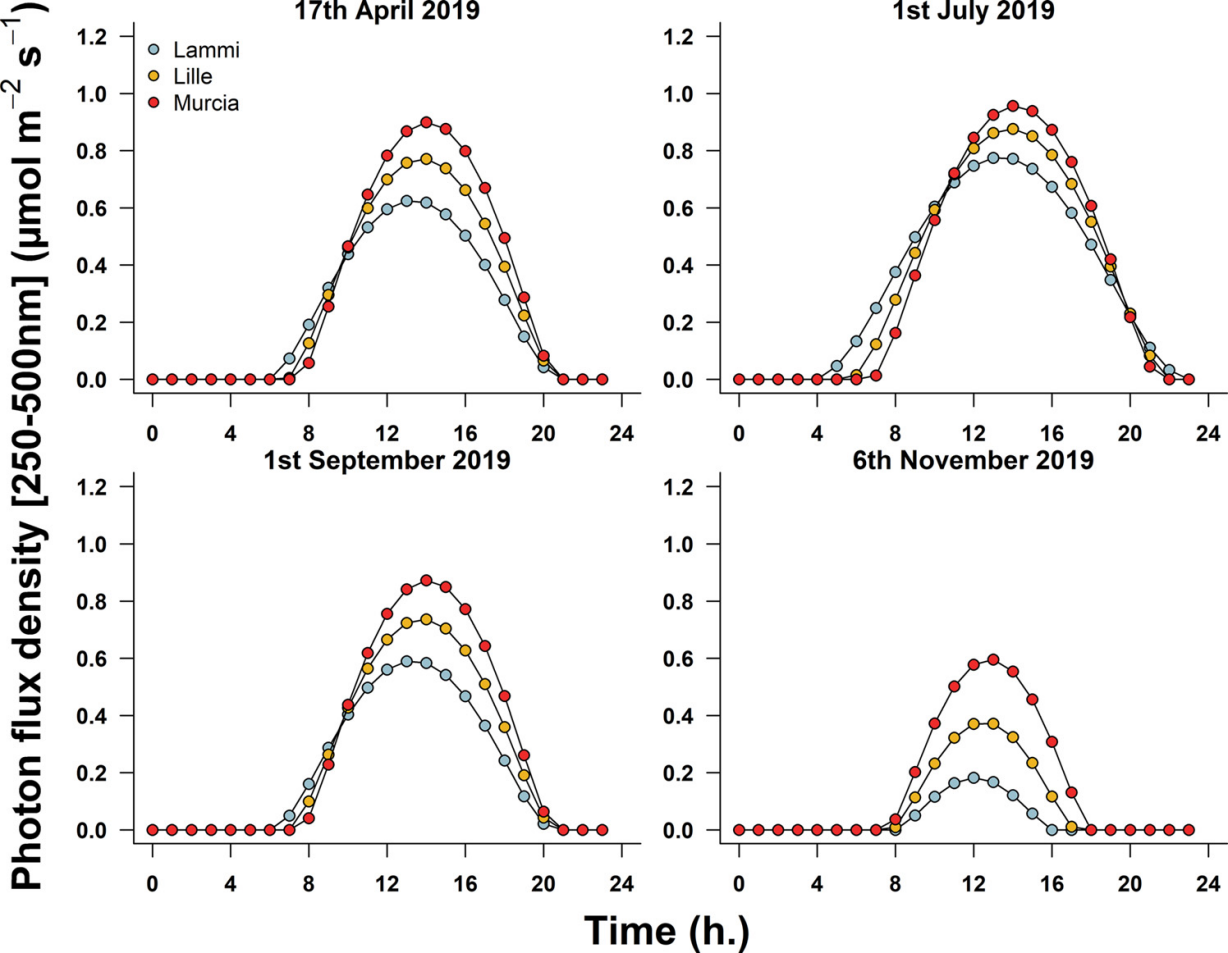
Diurnal variations in photon flux density (between 250 and 500 nm wavelength) incident on a sensor (*e.g.* an eye) looking at 15° below the horizon and at 170 cm above the ground, from three locations (Lammi, Finland; Lille, France; Murcia, Spain), four dates (April 17th, July 1st, September 1st, and November 6th 2019), and for an aerosol optical density of 0.1. Simulations were parametrized according to local meteorological condition (elevation, pressure, temperature) and atmospheric conditions on the simulated day (water column, O_3_ and NO_2_ concentrations). Data were averaged over eight cardinal directions, and normalized to a field of view of 17°.

**Fig. 3:**
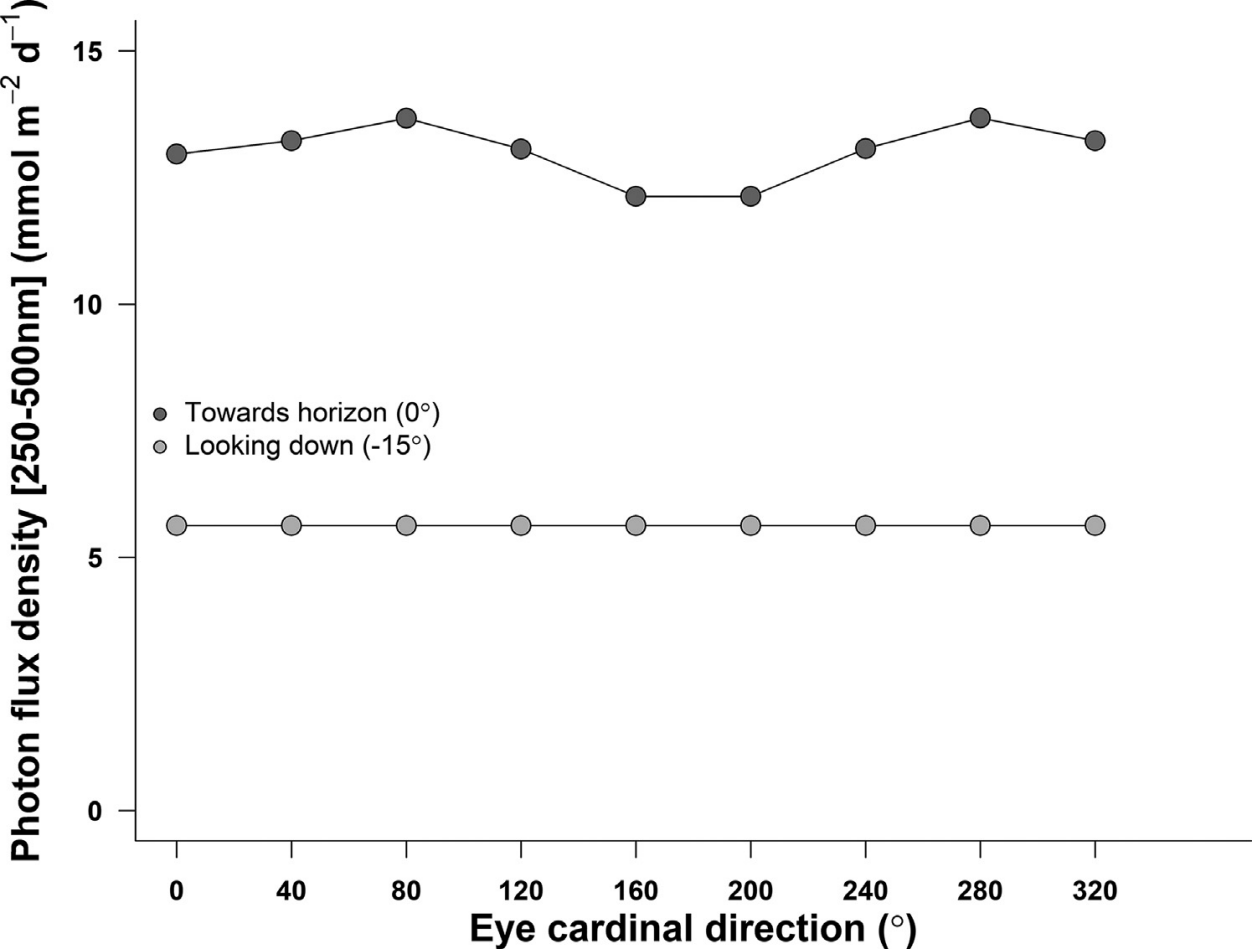
Photon flux density (between 250 and 500 nm wavelength) incident on a sensor (*e.g.* an eye) looking either towards the horizon (dark gray) or at 15° below it (light gray), in relation to the eye cardinal direction. Data was modeled for an urban soil surface, on the 1st of July 2019, and with the sensor at 170 cm above the ground. Simulations were parametrized according to local meteorological conditions (elevation, pressure, temperature) and atmospheric conditions on the simulated day (water column, O_3_ and NO_2_ concentrations). Data were normalized to a field of view of 17°.

**Fig. 4:**
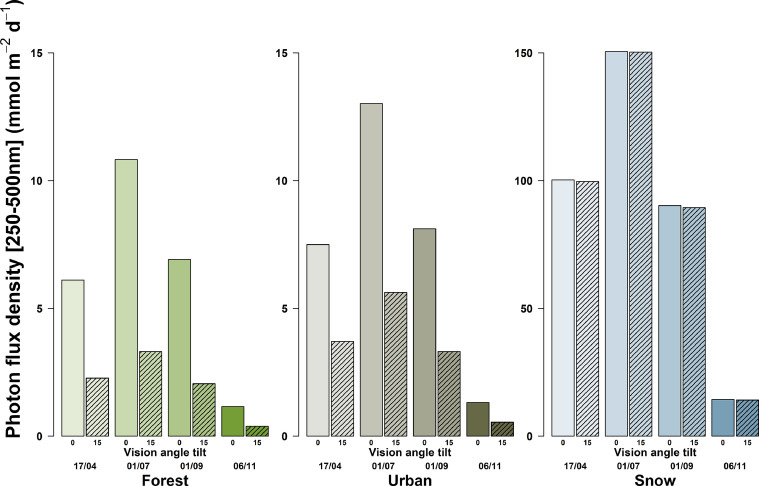
Total daily photon flux density (between 250 and 500 nm wavelength) incident on a sensor (*e.g.* an eye) looking either towards the horizon or at 15° below it, and at 170 cm above the ground, from four dates (April 17th, July 1st, September 1st, and November 6th 2019), and three soil surface types (forest, urban and snow). Simulations were parametrized according to local meteorological condition (elevation, pressure, temperature) and atmospheric conditions in Lammi (Finland) on the simulated day (water column, O_3_ and NO_2_ concentrations). Data were averaged over eight cardinal direction, and normalized to a field of view of 17°.

**Fig. 5:**
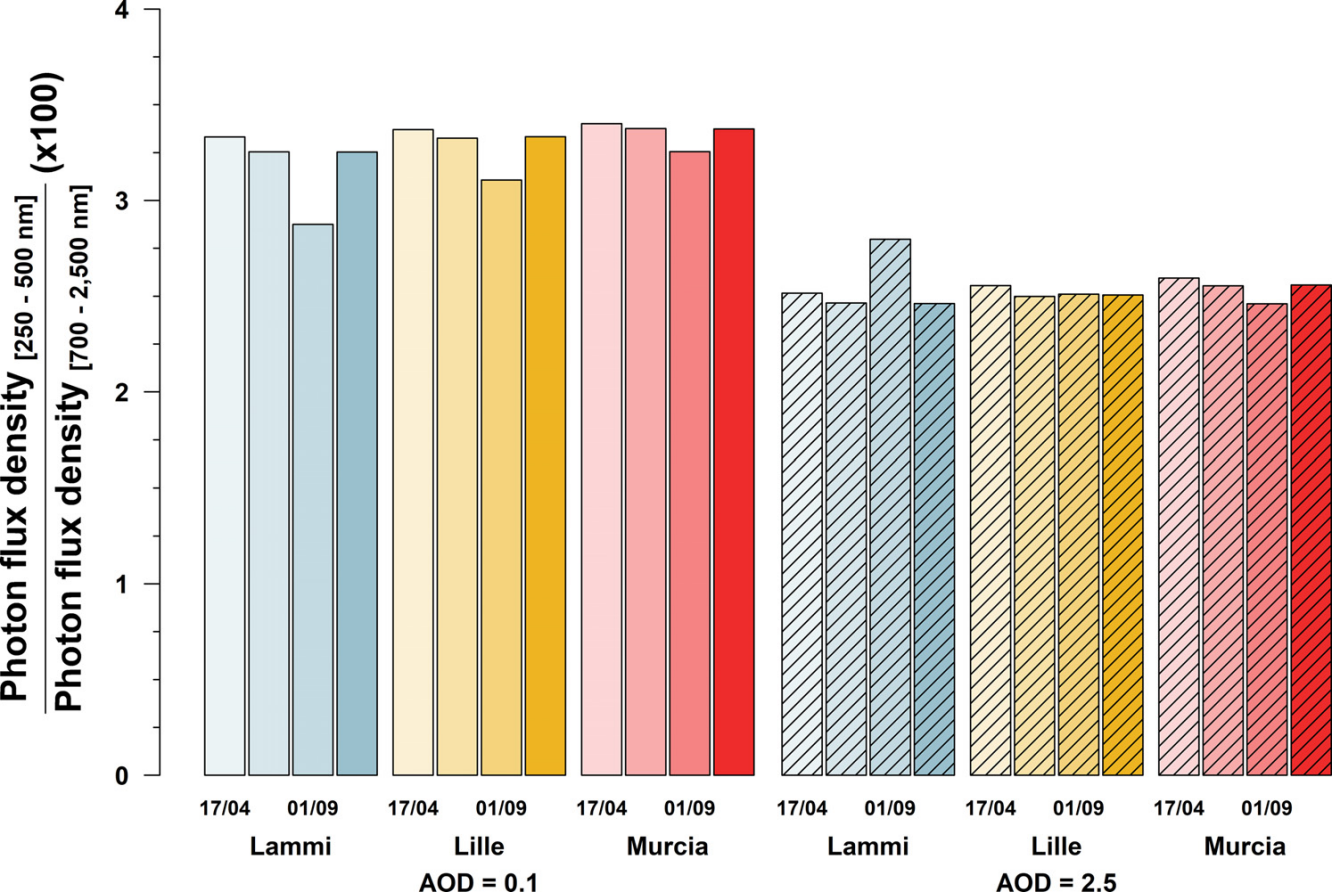
Ratio of daily photon flux density in the shortwave (250–500 nm wavelength) to that in the infrared (700 – 2500 nm) incident on a sensor (e.g. an eye) looking northward at 15° below the horizon, and at 170 cm above the ground, from three locations (Lammi, Finland; Lille, France; Murcia, Spain), four dates (April 17th, July 1st, September 1st, and November 6th 2019), and two aerosol optical densities (AOD). Simulations were parametrized according to local meteorological condition (altitude, pressure, temperature) and atmospheric conditions on the simulated day (water column, O_3_ and NO_2_ concentrations). Data were normalized to a field of view of 17°.

**Fig. 6:**
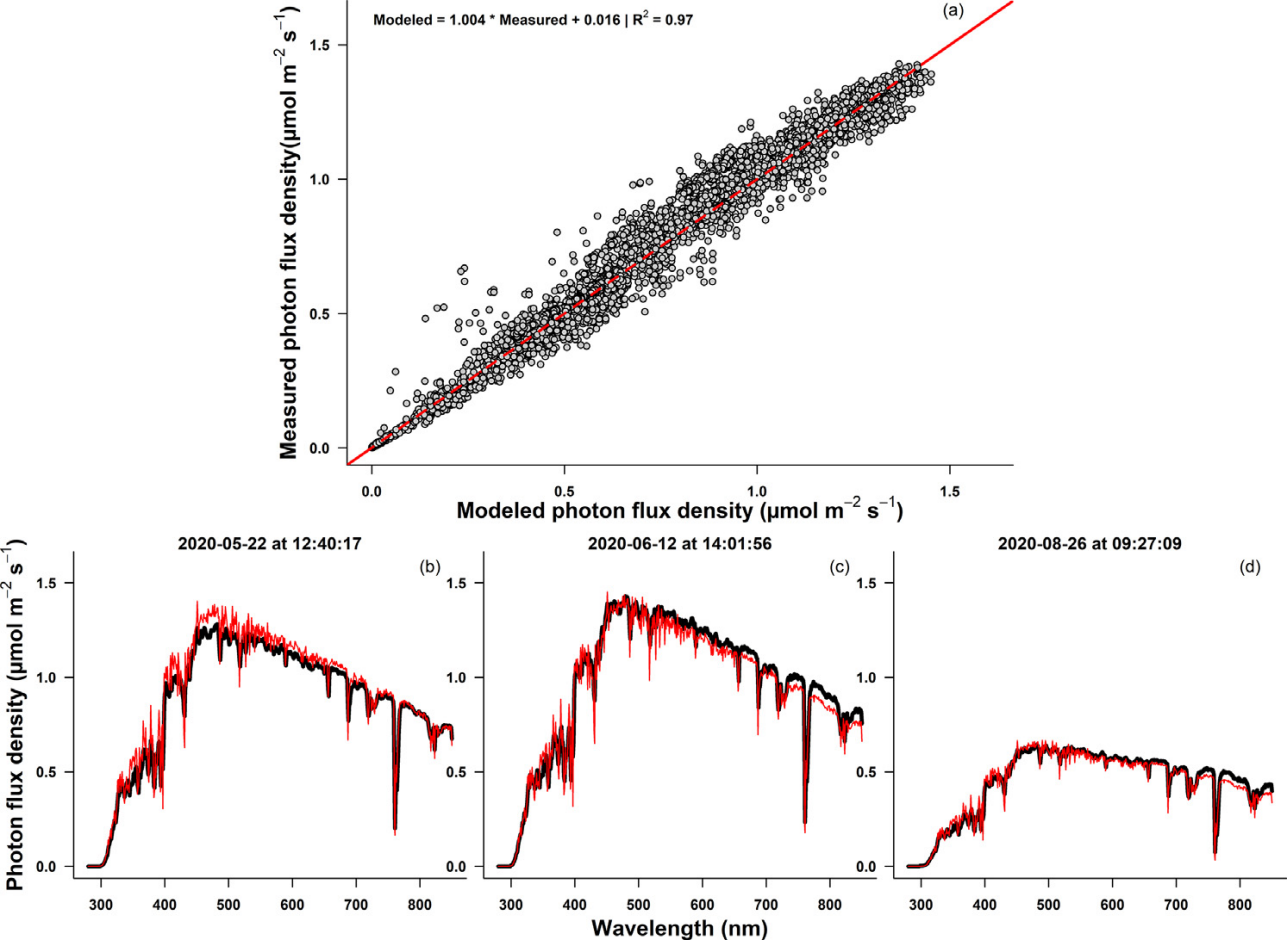
Comparison of modeled and measured photon flux density in Lammi, Finland. (a) Concordance between modeled and measured data. Each point represent a wavelength between 280 and 850 nm from nine measurements done between May 22nd and August 26th 2020. Coefficients and R^2^ for the linear regression between modeled and measured data is given on the top left. The identity line is shown in red. (b–d). Example comparisons of measured (in black) and modeled (in red) photon flux density. Spectra were chosen to illustrate a range of diurnal and seasonal variability in photon flux density.

Figures show comparisons between the different conditions modeled in each simulation. The effect of aerosol optical density on the total radiation (250–500 nm) incident on the eye for each date and location is presented in Fig. 1, and similarly for diurnal variations in Fig. 2. Fig. 3 shows how the cardinal direction and tilt of the viewing angle affects incident radiation on the eye, while Fig. 4 shows the interaction of surface reflectance and tilt of the viewing angle, at each date. Fig. 5 highlights differences in the ratio of shortwave (250–500 nm) to infrared wavelengths (700–2500 nm) incident on the eye. Additionally, Fig. 6 shows a comparison of measured and modeled photon flux density in Lammi, Finland. Measurements were done in an open area, using a CCD array spectroradiometer Maya 2000 Pro (Ocean Optics, Dunedin, FL, USA) attached to a cosine diffuser (D7-H-SMA, Bentham Instruments Ltd., Reading, UK). For more information about the measurements, please see [7], [8], [9]. Modeled data for Fig. 6 was simulated over the 280–850 nm range, at the exact date, time, and location of the measured data. The soil surface was of a forest floor, with aerosol optical density retrieved from the satellite AERONET network. All other parameters were the same as those used in the simulations 1 to 3 presented in Fig. 1 to 5.

## 3 Experimental Design, Materials and Methods

### 3.1 Model Parametrization

We used the libRadtran (v2.0.3) radiative transfer model [10,11] because of its use in a variety of environmental conditions to model sunlight spectral radiation, such as for comparing patterns in the spectral composition between Helsinki (Finland) and Gual Pahari (India) [12]. We used the discrete ordinate solver “disort”, with six streams and delta-M scaling. For absorption parametrization, the LOWTRAN spectral calculations were used. The parametrization of the atmospheric profile was taken from the standard atmosphere for mid-latitudes in summer, provided with the model. It describes the atmospheric profile of pressure, temperature, air density, O_3_, O_2_, H_2_O, CO_2_ and NO_2_. O_3_, and NO_2_ were then scaled using satellite data from the AERONET network [13] at each day and locations modeled, because of large regional and seasonal trends. H_2_O concentration was scaled from a value of 10 kg *m* ^−^ ^2^, because of its large day-to-day variation, and to improve the comparison between simulations. The output radiances (mW *m* ^−^ ^2^ nm^−1^ sr^−1^) have a resolution of 1 nm.

Three locations were selected, on a latitudinal gradient, in southern Finland (Lammi; 61.054 N, 25.042 E), northern France (Lille; 50.612 N, 3.142 E) and southern Spain (Murcia; 38.001 N, 1.171 S)*.* Elevations and coordinates were specific to each location. Four dates were chosen, based on the availability of satellite data from the AERONET network at the three locations and representing each season of the year 2019. Data from the winter were often missing, so the April 17th (DOY 107), July 1st (DOY 182), September 1st (DOY 244) and November 6th (DOY 310) were selected.

To obtain better comparisons between locations, the same aerosol-specific parameters were used for all simulations. We used a pre-defined [14] spring-summer profile, 50 km visibility, with rural type aerosols in the lower 2 km of the atmosphere, and background volcanic aerosols above 2 km. Unless specified, the aerosol optical density was set as 0.1 at 380 nm, and other wavelengths were scaled accordingly by the model. The soil surface type was selected from a collection of spectral albedos in the built-in International Geosphere Biosphere Programme library and was set to “urban” unless otherwise specified. The detector was set as 170 cm above the ground (*i.e.* average human height), facing various directions (see Table 1).

### 3.2 Batch Processing

The libRadtran model was installed on a Windows 10 operating system *via* the Cygwin GNU collection (v3.1.4). A script in R (v4.0.3) was written to automatically read the set of input parameters for each calculation from a summary .csv file (please refer to Table 1 for a list of the variable input parameters). Then, an input file was created by modifying a template according to the requested set of input parameters for the given calculation. A command script (.cmd) was also created to run libRadtran *via* Cygwin with the given input file. After the calculations were done, the output file was formatted for further analyses into a .txt file, and assigned a unique name based on the specific input parameters used in the calculation.

For the figures, the output radiances were scaled for the field of view relevant to the macula (∼17° [5],) using the formula:

Ω=2π(1−cosθ)

with Ω the scale factor, and θ the radius of the field of view (8.5°). Finally, radiances were converted to µmol m^−2^ s^−1^ using the “*photobiology*” R packages [15].

## Ethics Statement

The dataset does not involve any human or animal subjects, nor has it been collected from social media platforms.

## CRediT authorship contribution statement

**Maxime Durand:** Methodology, Software, Formal analysis, Data curation, Visualization, Investigation, Writing – original draft, Writing – review & editing. **Andrew McLeod:** Conceptualization, Writing – review & editing.

## Declaration of Competing Interest

The authors declare that they have no known competing financial interests or personal relationships that could have appeared to influence the work reported in this paper.

## Data Availability

A dataset of global variations in directional solar radiation exposure for ocular research using the libRadtran radiative transfer model (Original data) (Zenodo).
